# Comprehensive Identification of the Bovine KLF Gene Family and Its Functional Regulation in Muscle Development: Insights from Single-Nuclei Transcriptomics

**DOI:** 10.3390/ani15192930

**Published:** 2025-10-09

**Authors:** Fengying Ma, Le Zhou, Lili Guo, Chencheng Chang, Dan Dan, Yanchun Bao, Guiting Han, Mingjuan Gu, Lin Zhu, Risu Na, Caixia Shi, Jiaxin Zhang, Wenguang Zhang

**Affiliations:** 1 College of Animal Science, Inner Mongolia Agricultural University, Hohhot 010018, China; fengyingma1997@163.com (F.M.); zxcvbnm8880314@163.com (L.Z.); changchencheng8112@163.com (C.C.); byc@emails.imau.edu.cn (Y.B.); 15934210949@163.com (G.H.); gmj0119@yeah.net (M.G.); zhulinynacxhs@163.com (L.Z.); narsanjargal@imau.edu.cn (R.N.); caixiashi@imau.edu.cn (C.S.); 2 Key Laboratory of Animal Genetics, Breeding and Reproduction of the Inner Mongolia Autonomous Region, College of Animal Science, Inner Mongolia Agricultural University, Hohhot 010018, China; 3 College of Life Science, Inner Mongolia Agricultural University, Hohhot 010018, China; 13474912747@163.com; 4 Rural and Pastoral Economic Service Center of Horqin Left Rear Banner, Tongliao 028000, China; dandan@emails.imau.edu.cn

**Keywords:** *Bos taurus*, Krüppel-like factors, longissimus dorsi, single-nucleus RNA sequencing, SHAP

## Abstract

The Krüppel-like factor (*KLF*) family of transcription regulators plays pivotal roles in adipogenesis, myogenesis, and metabolism. While comprehensively studied in humans and mice, its characterization in cattle remains limited, especially within the skeletal muscle niche. This study aimed to systematically characterize the *KLF* family in Bos taurus and elucidate its role in breed-specific muscular development. We employed an integrated approach of comparative genomics and single-nucleus RNA sequencing (snRNA-seq) on longissimus dorsi muscle from Angus (ANG, beef breed) and Holstein (HST, dairy breed) cattle. Phylogenomic analysis identified 14 *KLF* genes, revealing evolutionary conservation and potential functional divergence. snRNA-seq delineated 11 distinct cell populations and uncovered cell-type-specific expression patterns of *KLFs*. Further machine learning based analysis pinpointed *KLF6*, *KLF9*, *KLF10*, and *KLF12* as key global drivers of transcriptional differences between breeds, while *KLF6* was identified as a major cell-type-specific contributor in lymphatic endothelial cells. Our work provides a foundational resource for understanding the *KLF* family in cattle and identifies promising candidate genes for improving meat production traits through molecular breeding.

## Simple Summary

Beef production is important for global food security. Understanding the genetic factors that control muscle growth and meat quality in cattle is key to improving breeding strategies. This study focused on a family of genes called *KLF* transcription factors, which are known to be master regulators of growth and metabolism. We systematically identified 14 *KLF* genes in the cattle genome. Using advanced single-nuclei RNA sequencing technology, we mapped the activity of these genes across different cell types (such as muscle fibers, fat cells, and immune cells) in the muscle tissue of two cattle breeds: Angus (beef breed) and Holstein (dairy breed). We discovered that the activity of these *KLF* genes varies greatly between cell types and between breeds. Using a machine learning approach, we identified *KLF6*, *KLF9*, *KLF10*, and *KLF12* as key genes that may drive the differences in muscle development between the two breeds. Our findings provide valuable genetic targets for future breeding programs aimed at enhancing beef quality and yield.

## 1. Introduction

Beef is a key source of high-quality protein and essential nutrients (such as iron, zinc, and vitamin B12) and occupies a central position in the global dietary structure [1]. With population growth and rising living standards, global demand for beef continues to climb. Therefore, ensuring the stability of beef production and improving its yield and quality are crucial to meeting market demand and promoting social and economic development.

In cattle production, biological and pathological factors (such as diseases affecting health and growth) and genetic traits (determining growth potential, muscle fiber type, and fat distribution) jointly influence skeletal muscle growth and development and fat deposition efficiency [2]. Molecular breeding technology, with its advantages of precisely improving genetic traits and simultaneously enhancing meat yield and quality, has become a hot topic in cattle genetic improvement research.

Skeletal muscle, as the main component of beef, not only plays a key role in the movement and metabolism of cattle, but also serves as the foundation of meat production [3]. However, muscle growth and development are precisely controlled by numerous genes and their regulatory networks, making it a highly complex biological process. The Krüppel-like factor (*KLF*) family comprises a group of fundamental transcription regulators whose members mediate various important biological functions through a DNA-binding domain containing three zinc finger structures [4]. A total of 18 *KLF* genes, designated *KLF1* through *KLF18*, have been identified in the human genome [5]. Research has shown that *KLF* genes have diverse functions in different species: for example, *KLF4*, *KLF2*, *KLF3*, and *KLF6* are highly expressed in monocytes, macrophages, and dendritic cells in mice, while *KLF1* is essential for red blood cell development [6]. In pig and human studies, *KLF* members are also involved in regulating muscle growth, metabolic processes, and cell proliferation and migration [7,8,9]. The role of the *KLF* gene in adipogenesis has been extensively studied. *KLF2* has been shown to be a key negative regulator of adipogenesis. Recently, a study on single cells showed that overexpression of *KLF2* in 3T3-L1 cells can inhibit *PPARγ*, thereby significantly reducing intracellular lipid accumulation [10]. *KLF3* is a member of the KLF family, primarily responsible for adipocyte differentiation and fat deposition. Studies have shown that miR-32-5p can affect fat production by downregulating *KLF3* in adipose tissue and intramuscular fat [11]. Due to the knockout of *KLF3* by siRNAs, bovine fat production can be inhibited [12]. There are also studies indicating that *KLF3* can inhibit the differentiation of porcine adipocytes in vitro by downregulating adipogenesis markers (including *PPARG*, *C/EBPA*, and *FABP4*) [11]. *KLF6* is an important transcription factor in skeletal muscle development [13]. Research indicates that the *KLF6* gene may serve as a potential candidate marker gene for beef breed improvement in QinChuan cattle through marker-assisted selection [14]. Research has shown that *KLF9* is a novel regulatory factor induced by glucocorticoids (GC) in skeletal muscle. Its function is directly related to muscle atrophy: overexpression of *KLF9* induces muscle atrophy, while knockout of its expression promotes muscle hypertrophy. These findings establish the position of *KLF9* as a key regulator of skeletal muscle mass and reveal its important role in the GC-induced muscle atrophy pathway [15]. *KLF4*, *KLF5*, *KLF6*, *KLF7*, *KLF10*, and *KLF15* are implicated in regulating skeletal muscle growth and development in mice [5,8,16,17,18], strongly suggesting that this family also plays a key role in muscle development in cattle. Although the *KLF* family has been systematically identified and functionally studied in other species (such as pigs and mice), there is still a significant lack of research on the bovine *KLF* gene family.

Given the established roles of *KLF6*, *KLF9*, *KLF10*, and *KLF12* in muscle and fat production in other mammals, we hypothesize that these key *KLF* members are the core regulatory factors of transcriptional differences in the skeletal muscle microenvironment between beef and dairy breeds, thereby affecting muscle phenotype and meat production traits.

Due to the consistency of skeletal muscle tissue, which is a complex microenvironment composed of multiple cell types, most existing studies have been conducted using Bulk RNA-seq, which cannot reveal the precise expression profile and functional differentiation of *KLF* genes in specific cell types. The emergence of single-cell transcriptome sequencing (scRNA-seq or snRNA-seq) technology has provided an unprecedented powerful tool for deconstructing tissue heterogeneity at the single-cell level, mapping cell-type-specific expression profiles, and analyzing gene regulatory networks [19,20]. There are significant differences in muscle growth rate and fat deposition among different cattle breeds, such as Angus (ANG) and Holstein (HST), which are, respectively, beef and dairy breeds. However, the cellular-level molecular mechanisms driving these differences, particularly the contribution of the *KLF* family, have not yet been revealed at single-cell resolution.

This study aims to systematically elucidate the regulatory mechanisms of the bovine *KLF* transcription factor family in the development of the longissimus dorsi (LD) and the formation of breed differences through the integration of multi-omics strategies. The study first conducted a comprehensive identification and evolutionary analysis of the bovine *KLF* family, elucidating its phylogenetic relationships, structural characteristics, and genomic distribution patterns; subsequently, using snRNA-seq data, it mapped the expression profiles of *KLF* genes in different cell types of the LD in ANG and HST cattle, revealing their expression heterogeneity and breed-specific differences. Finally, combining machine learning and SHAP analysis, the study identified key *KLF* genes influencing muscle phenotype and inferred their regulatory networks. This study is the first to elucidate the functional mechanisms of the bovine *KLF* family at the single-nuclei level, providing important theoretical foundations for understanding the molecular basis of muscle development and molecular breeding in beef cattle.

## 2. Materials and Methods

### 2.1. Identification of KLF Family

To systematically identify and characterize the *KLF* gene family in bovine (*Bos taurus*) and enable comparative evolutionary analysis, we retrieved protein sequences from four representative mammalian species: *Homo sapiens*, *Mus musculus*, *Sus scrofa*, and *Bos taurus*. All sequences were downloaded from the UniProtKB database (Release 2023_08) using taxon-specific filters and the query term “Krüppel-like factor”. Only manually reviewed entries were retained. For each gene, the longest isoform encoding a complete DNA-binding domain was selected to ensure consistency and representativeness across species.

This process identified 18 *KLF* members in *Homo sapiens*, 17 in *Mus musculus*, 13 in *Sus scrofa*, and 14 in *Bos taurus*. The complete list of UniProt accession IDs for all *KLF* orthologs analyzed in this study is provided in Table S2. To further ensure the accuracy and reliability of the bovine *KLF* family set, all candidate sequences were validated by confirming the presence of the characteristic triple C_2_H_2_ zinc finger domain using Pfam domain analysis. Chromosomal locations and gene annotations for bovine *KLFs* were based on the ARS-UCD1.2 genome assembly and orthology information from human *KLF* nomenclature. The final high-confidence, multi-species KLF protein sequence dataset was used for subsequent phylogenetic reconstruction.

### 2.2. Structural and Evolutionary Analysis of KLF Genes: Protein Alignment, Phylogeny, and Chromosomal Location

To reconstruct the phylogeny of *KLF* genes, a multiple sequence alignment of all identified orthologs from the four species was generated using the MUSCLE algorithm [21].

To conduct this study, longissimus dorsi (LD) muscle samples were harvested from 6 ANG and 6 HST cattle provided by Xuyi Animal Husbandry Co., Ltd., Bayannur City, Inner Mongolia Autonomous Region, China. All animals were healthy adult males with similar age (36 months) and body weight (550–600 kg), raised under standardized feeding conditions. Tissue samples were harvested immediately post-mortem by trained personnel using aseptic techniques. For RNA integrity preservation, samples were flash-frozen in liquid nitrogen within 5 min of collection and stored at −80 °C until further processing. All animal procedures were approved by the Animal Ethics Committee of Inner Mongolia Agricultural University (Approval No. DK20231115; Date: 15 November 2023).

### 2.3. Isolation of Nuclei from Bovine LD Tissue

snRNA-seq was performed by personnel from MGI Tech Co., Ltd. (Shenzhen, China) Laboratory using the DNBelab C Series Single-Cell Library Prep Kit (TaiM 4, No. [940-001818-00,16RXN]) following the manufacturer’s protocol. The procedure involved: (1) droplet-based encapsulation of single nuclei with barcoded gel beads [22], (2) reverse transcription within droplets to generate barcoded cDNA, (3) cDNA amplification and purification, and (4) library construction for sequencing. LD samples were surgically extracted and rapidly frozen in liquid nitrogen. The intact nuclei were then isolated and purified with modifications to standard protocols. Briefly, the frozen tissue was minced into approximately 5 mm × 5 mm pieces and homogenized on ice in Nuclei EZ Lysis Buffer (Sigma-Aldrich, St. Louis, MO, USA; Catalog No. NUC101) for 10 min. The homogenate was filtered through a 40-μm cell strainer, and the nuclear suspension was centrifuged at 500× *g* for 5 min at 4 °C [23]. The pellet was resuspended in PBS to concentrate the nuclei. Nuclear integrity and concentration were assessed under a fluorescent microscope. Finally, the nuclear concentration was adjusted to approximately 1000 nuclei per microliter for library preparation [24].

### 2.4. Preparation of Single-Nuclei Libraries and Sequencing

The snRNA-seq libraries were prepared with the DNBelab C4 Single-nucleus RNA Library Prep Kit (MGI Tech Co., Ltd.) following the vendor’s protocol (DNBelab C User Manual V3.0) [25]. Briefly, isolated nuclei were first encapsulated in barcoded gel beads using the DNBelab C4 instrument to form Gel Bead in-emulsion (GEM) reactions. After emulsion breaking (performed with Recovery Agent, MGI), the released beads carrying 10 bp cell-barcodes (CB1 and CB2) and a 10 bp UMI were collected and subjected to reverse transcription (RT) at 42 °C for 90 min using RT Enzyme Mix (MGI). Second-strand synthesis was followed by 14 cycles of cDNA amplification with Amplification Enzyme Mix (MGI). Amplified cDNA was purified twice with 0.6× DNBelab Clean Beads, and 200–400 bp fragments were size-selected for library construction. Indexed sequencing libraries were generated with the DNBelab C4 Library Prep Module (MGI) using 10–12 cycles of PCR and purified again with 0.8× Clean Beads. Final libraries were quantified on a Qubit 4 fluorometer with the Qubit ssDNA Assay Kit (Invitrogen, Waltham, MA, USA; Cat. No. Q10212) and verified on a 2100 Bioanalyzer (Agilent Technologies, Santa Clara, CA, USA) [26]. Paired-end sequencing (read-1 = 30 bp, read-2 = 100 bp) was performed on a DNBSEQ-T7 platform at China National GeneBank (Shenzhen, China). All experimental steps were carried out according to the manufacturer’s instructions (DNBelab C User Manual V3.0; MGI, 2025).

### 2.5. Single-Nucleus Data Analysis

Raw sequencing data were processed using the DNBC4tools (v2.1.0) specific to the MGI DNBelab C platform. First, a custom reference genome index was built using the “DNBC4tools mkref” function with the ARS-UCD1.2 bovine genome assembly. The pipeline was then applied to demultiplex raw data, extract cellular barcodes and UMIs, and generate the single-nuclei gene expression matrix. Subsequent analysis was performed in Seurat (v4.4.0) [27] for quality control, normalization, and cell clustering. Cells were filtered based on the following thresholds: nFeature_RNA > 200, nCount_RNA < 3000, and percent.mt < 10%. Following filtering, a total of 59,563 nuclei from ANG cattle and 51,835 nuclei from HST cattle were retained for downstream analysis. Table S1 provides a summary of sequencing and quality metrics for each sample, including total reads, mean reads per nucleus, mapping rate, and median genes per nucleus. The data were normalized using the NormalizeData function in Seurat with the default normalization method (“LogNormalize”) and a scale factor of 10,000. To mitigate batch effects, we applied Harmony [28] integration using the RunHarmony function with default parameters and the batch covariate set to ‘orig.ident’. Scaling and PCA were performed using the ScaleData function (regressing out the nCount_RNA and percent.mt variables) and RunPCA function, (using the top 2000 variable features and the first 50 principal components for downstream analysis), regressing out effects of UMI count per cell and mitochondrial content. For visualization and clustering, we conducted uniform manifold approximation and projection (UMAP) based on the first 50 principal components using the RunUMAP function. Cell clusters were identified using the FindClusters function with a resolution parameter of 0.2 and the Leiden algorithm. To identify marker genes across clusters or between conditions, differential expression analysis was performed using the FindAllMarkers function in Seurat, which implements the Wilcoxon rank sum test. This function provides two key statistical outputs for each gene: a probability value (*p*-value) adjusted for multiple testing using the Bonferroni correction (default in Seurat) to control the family-wise error rate (FWER), and an effect size measure, reported as the area under the curve (AUC). The AUC quantifies the discriminative power of the gene, representing the probability that a randomly selected cell in the target cluster has a higher expression level than a cell in other clusters (where 0.5 indicates no discriminative ability, and 1.0 or 0.0 indicates perfect separation). Differentially expressed genes (DEGs) were defined as those meeting a Bonferroni-adjusted *p*-value < 0.05, an absolute log_2_ fold change |log_2_FC| > 0.25, a minimum percentage of expressing cells (min.pct) > 0.1, and an effect size threshold of |AUC − 0.5| > 0.1 to ensure biological relevance beyond statistical significance.

### 2.6. Cell Type Annotation

We first used scMayoMap [29] software to automatically annotate single-nuclei data to preliminarily identify possible cell types for each cluster. Subsequently, to further improve the accuracy of annotations, we adopted manual annotations as a supplementary strategy. Specifically, we systematically summarized marker genes for muscle cell types from published literature [30]. Next, we developed a specialized R script to compare and integrate the automatically annotated results with manually collected muscle cell marker genes. The specific operation is to obtain the intersection of the top 25 marker genes in each cluster and the muscle cell type marker genes in the literature, in order to determine more accurate candidate cell types. We normalized the average log_2_FC values of each marker gene for each candidate cell type in the intersection set to individual scores, and added up the scores of all marker genes as a comprehensive score to evaluate the likelihood of each cell type. A high score means that the cluster is more likely to belong to the corresponding cell type. Finally, by integrating previous research reports, we ultimately determined the cell type.

### 2.7. Explainable Machine Learning

To quantify the regulatory significance of the 14 *KLF* genes in distinguishing cellular identities and breed-specific expression patterns, we trained an XGBoost classifier on snRNA-seq data from bovine LD. To quantify the regulatory significance of the 14 *KLF* genes in distinguishing cellular identities and breed-specific expression patterns, we trained an XGBoost classifier on snRNA-seq data from bovine LD. SHAP (SHapley Additive exPlanations) values were computed to quantify the effect size and direction of each gene’s contribution to the model’s prediction. The mean absolute SHAP value for each gene was used to rank its global feature importance, while the sign and magnitude of individual SHAP values were used to interpret the direction and strength of its effect in specific cellular contexts. Model performance was evaluated using the area under the receiver operating characteristic curve (AUC = 0.705) with 95% confidence intervals derived from bootstrapping (*n* = 1000). The AUC metric was prioritized due to its robustness to class imbalance and focus on overall discriminatory power, aligning with the study’s goal of identifying genes with cross-condition predictive utility. Gene rankings were validated through repeated training with different random seeds, and only genes with consistent SHAP-based contributions across iterations were considered robust and reported in the final interpretation of results.

## 3. Results

### 3.1. Characterization and Evolutionary Analysis of the KLF Gene Family

**Figure 1:**
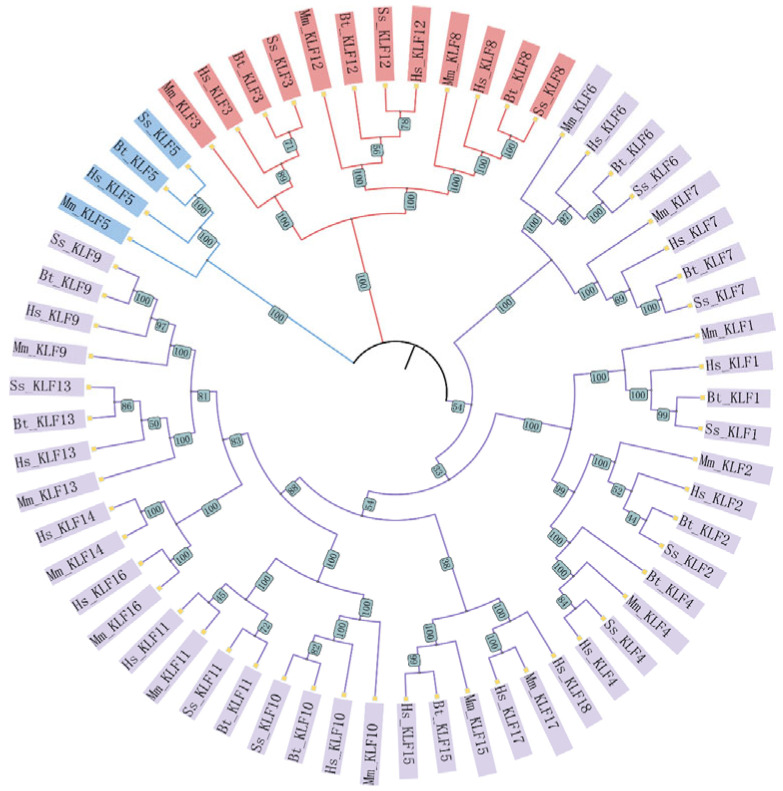
Phylogenetic analysis of KLF proteins in *H. sapiens*, *M. musculus*, *S. scrofa* and *B. taurus*. A neighbor-joining phylogenetic tree was constructed from the sequences of the 62 proteins and categorized into three clades (I–III). Species abbreviations are as follows: Hs (*Homo sapiens*); Mm (*Mus musculus*); Ss (*Sus scrofa*); Bt (*Bos taurus*).

A total of 62 *KLF* genes were identified across four species based on the presence of the conserved zf-H2C2 domain: 18 in *Homo sapiens* (Hs), 17 in *Mus musculus* (Mm), 13 in *Sus scrofa* (Ss), and 14 in *Bos taurus* (Bt); the protein sequence is shown in Table S3. To investigate the evolutionary relationships among *KLF* family members, an NJ phylogenetic tree was constructed using protein sequences of these 62 genes (Figure 1). Phylogenetic analysis classified the genes into three distinct groups (I–III) based on sequence homology and branching patterns. Group I (red, 12 genes) contained orthologs such as *Mm-KLF3/12/8*, *Hs-KLF3/12/8*, *Ss-KLF3/12/8*, and *Bt-KLF3/12/8*. Group II (blue, 4 genes) included *Mm-KLF5*, *Hs-KLF5*, *Ss-KLF5*, and *Bt-KLF5*. The remaining 46 genes formed Group III (purple), representing a larger divergent cluster.

### 3.2. Genomic Distribution of Bovine KLF Genes

**Figure 2:**
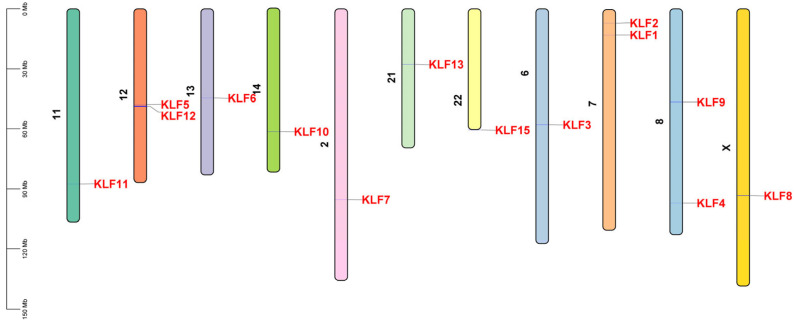
Chromosomal location of *KLF* genes on eleven chromosomes in bovines.

The 14 identified bovine *KLF* genes were unevenly distributed on 11 chromosomes (Figure 2). Chromosomes 7, 8, and 12 contain two *KLF* genes each, while the remaining 8 chromosomes contain one gene each. *KLF1* and *KLF2* are located on chromosome 7, *KLF4* and *KLF9* are located on chromosome 8, and *KLF5* and *KLF12* are located on chromosome 12. A single gene is located on chromosome 2 (*KLF7*), chromosome 6 (*KLF3*), chromosome 11 (*KLF11*), chromosome 13 (*KLF6*), chromosome 14 (*KLF10*), chromosome 21 (*KLF13*), chromosome 22 (*KLF15*), and chromosome X (*KLF8*). Table 1 provides detailed information on the genome coordinates, protein germplasm (UniProt), and coding sequences (CDS) of all 14 genes.

### 3.3. Analysis of Conserved Motifs and Protein Domains

**Figure 3:**
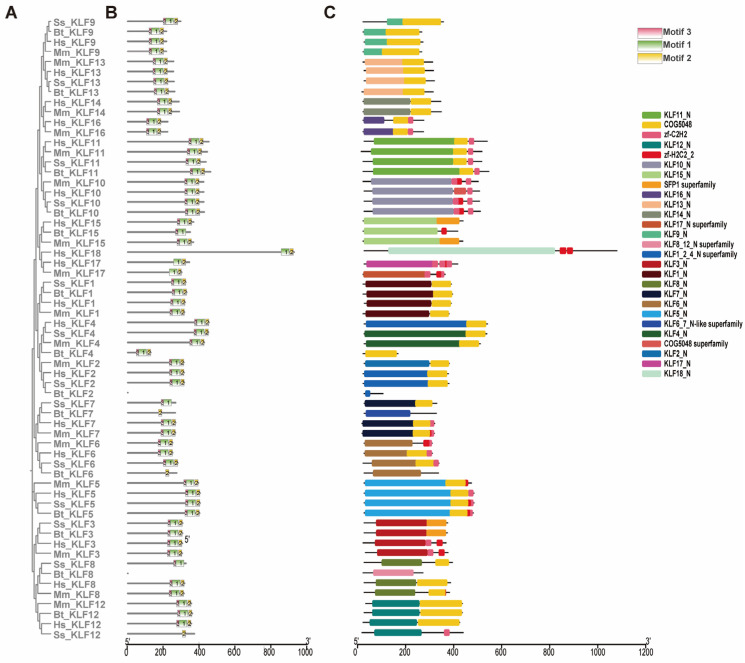
Phylogenetic relationships, Motif identification, and gene structure analysis. (**A**) Phylogenetic tree constructed by the neighbor-joining method, representing evolutionary relationships among 62 *KLF* genes across four species: *B. taurus*, *H. sapiens*, *M. musculus*, and *S. scrofa*. (**B**) Distribution of conserved protein motifs across the *KLF* gene family. Colored boxes represent distinct conserved motifs; gray lines correspond to non-conserved linker regions. (**C**) Conserved domain architecture of the *KLF* genes, illustrating functional protein domains shared across species.

An integrative approach combining phylogenetic reconstruction (Figure 3A) with analyses of gene architecture and conserved motifs was employed to elucidate the evolutionary relationships among *KLF* genes from *B. taurus*, *H. sapiens*, *M. musculus*, and *S. scrofa*. Through hidden Markov model (HMM)-based profiling, we identified three characteristic sequence motifs within the KLF protein family. Among these, Motif 1 and Motif 2 exhibited high conservation across most members. A notable exception was observed in bovine *KLF2* and *KLF8* (*Bt-KLF2/8*), which lacked all three motifs, implying a distinct evolutionary pathway for these genes (Figure 3B). Furthermore, closely related genes within the same phylogenetic subgroups displayed consistent motif patterns, suggesting potential functional similarities. Conserved domain analysis revealed 27 distinct protein domains across the 56 *KLF* genes examined (Figure 3C). Genes clustering within the same phylogenetic group demonstrated shared domain architectures, highlighting the structural conservation within subfamilies.

### 3.4. Analysis of Evolutionary Conservation and Functional Interaction Maps

**Figure 4:**
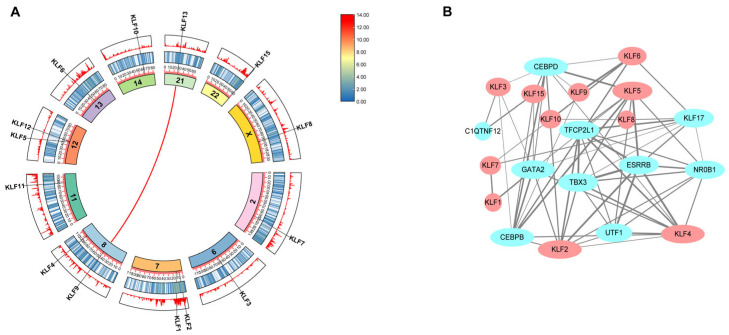
Synteny analysis and interaction network analysis. (**A**) Circos plot depicting genomic synteny of the *KLF* gene family across the bovine genome. The innermost ring represents the 11 chromosomes, while the middle and outer rings display gene density gradients via heatmap and linear plots (red: high density; white: medium; blue: low). *KLF* gene loci are annotated along the chromosomes. Colored arcs indicate collinear relationships between chromosomes, revealing conserved syntenic blocks involving *KLF* genes. (**B**) Protein–protein interaction network of *KLF* transcription factors. Nodes represent KLF proteins and their functionally associated partners.

Genomic distribution analysis (Figure 4A) revealed that all 14 bovine *KLF* genes exhibited low gene density in both middle and outer chromosomal regions. Notably, *Bt-KLF13* and *Bt-KLF9* displayed a syntenic relationship (red linkage), while no other *KLF* pairs showed collinearity. To explore functional associations, a protein–protein interaction (PPI) network was constructed (Figure 4B), identifying 11 KLF proteins with putative interactions. These interactions are connected to 10 functional genes (e.g., *CEBPB*, *UTF1*, *ESRRB*), suggesting potential regulatory crosstalk.

### 3.5. Prediction of the Tertiary Structure and Transmembrane Region of KLF Family Proteins

**Figure 5:**
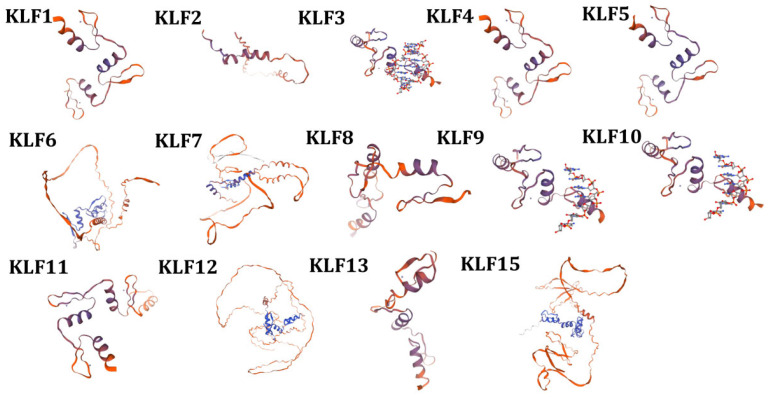
Prediction of protein tertiary structure of the bovine *KLF* gene family.

Homology modeling using the SWISS-MODEL server generated tertiary structure predictions for 14 bovine KLF proteins (Figure 5). The three-dimensional structure of proteins consists of motifs and domains. The activity and function of proteins are not only determined by the primary structure of protein molecules, but also closely related to their unique spatial structure. Incorrect spatial structure in proteins can lead to decreased protein function or even inactivation, which may also result in a series of mutations.

**Figure 6:**
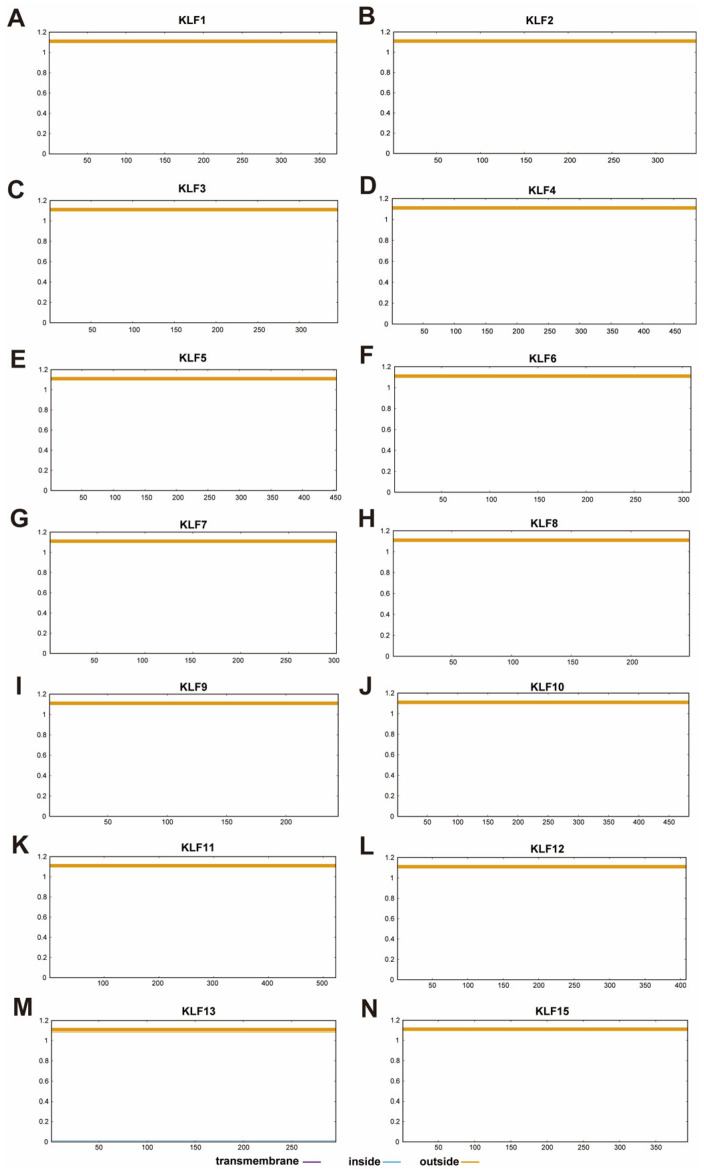
TMHMM prediction of transmembrane helices for bovine KLF proteins. TMHMM posterior probability for (**A**) KLF1, (**B**) KLF2, (**C**) KLF3, (**D**) KLF4, (**E**) KLF5, (**F**) KLF6, (**G**) KLF7, (**H**) KLF8, (**I**) KLF9, (**J**) KLF10, (**K**) KLF11, (**L**) KLF12, (**M**) KLF13, (**N**) KLF15; The Y-axis shows the posterior probability that each amino acid residue in the protein sequence belongs to one of three states: a transmembrane helix (purple line), the inside of the membrane (blue line), or the outside of the membrane (brown line). A probability value close to 1 indicates a high confidence prediction for that state at a given residue position. The X-axis represents the amino acid residue number along the protein sequence.

Transmembrane helix prediction was performed for all bovine KLF proteins using the TMHMM software (https://services.healthtech.dtu.dk/service.php? TMHMM2.0, accessed on 14 August 2025). Figure 6 shows the use of TMHMM to predict the transmembrane regions of 14 KLF protein sequences in bovine. The results show that none of the bovine KLF proteins contain transmembrane helical domains, as predicted by TMHMM. This finding is consistent with the well-established role of KLF transcription factors as intracellular proteins that function within the nucleus. The absence of transmembrane domains confirms that KLF proteins are not integral membrane proteins; however, subcellular localization (e.g., nuclear versus cytoplasmic) and potential secretion pathways would require further experimental validation using tools designed to predict signal peptides (e.g., SignalP) or nuclear localization signals (NLS).

### 3.6. Overview of KLF in the snRNA Seq Dataset of Bovine LD

**Figure 7:**
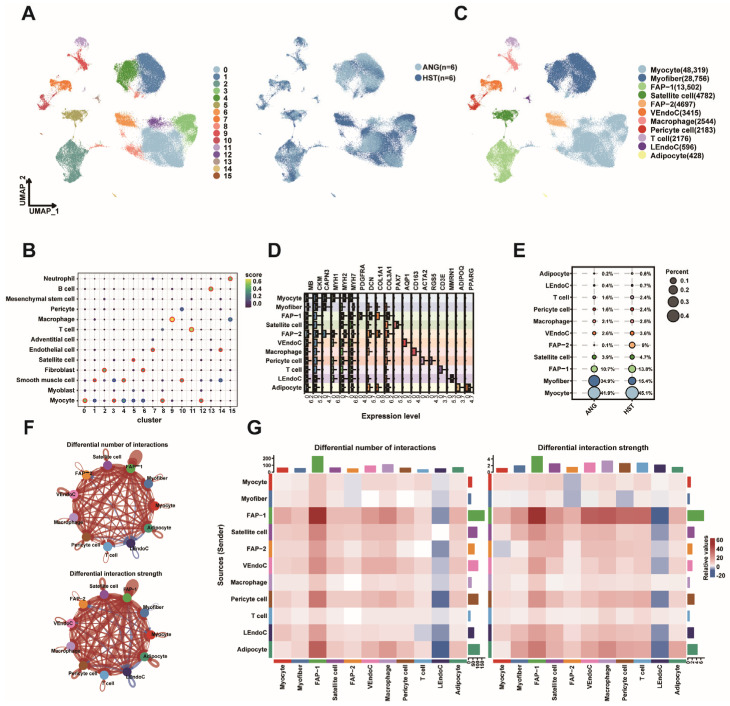
SnRNA-seq reveals multiple cell types in bovine LD. (**A**) UMAP graphs show unsupervised clustering of cells from all breeds identified by snRNA-seq. (**B**) The dot plot shows the automatic annotation of 16 cell clusters into 10 cell types using scMayoMap. (**C**) The UMAP graph shows the results annotated based on the Marker gene. (**D**) A box plot shows the expression of representative marker genes used in cell-type annotation. (**E**) The dot plot shows the proportion of each cell type in two breeds. (**F**) Comparison of the quantity and intensity of interaction between two types of LD cells. (**G**) Comparison diagram of the interaction between two breeds of LD cells. The left figure shows the number of interactions, and the right figure shows the strength of interactions between different cell types. The colored bar charts at the top and right represent the sum of values displayed in the heatmap. Red represents ANG stronger than HST, and blue represents HST stronger than ANG.

snRNA-seq was performed on LD samples from 3-year-old ANG and HST cattle to investigate the *KLF* gene family distribution. Following quality control, 111,398 cells were retained for downstream analysis (59,563 ANG; 51,835 HST). Unsupervised clustering identified 16 cell populations (Figure 7A), which were initially annotated into 9 cell types using the scMayoMap automated tool (Figure 7B). Manual refinement using established skeletal muscle markers (*Pax7* for satellite cells, *MYH1* and *MYH2* for myofibers) further resolved 11 distinct cell types (Figure 7C), with corresponding marker genes validated in Figure 7D [30]. Cell type composition analysis, based on six biological replicates per breed, revealed notable but statistically underpowered trends in breed-specific differences, likely due to high inter-individual variability (Figure 7E; Table S4). The most substantial trend was a higher proportion of myofiber in ANG cattle (mean ± SD: 32.9 ± 36.1%) compared to HST cattle (17.9 ± 21.2%), representing a large absolute difference (mean diff. = 15.0%, 95% CI: −24.3 to 54.4) with a medium effect size (Cohen’s d = 0.51), although this did not reach conventional statistical significance (*p* = 0.40). Conversely, multiple cell types—including FAP-1 (mean diff. = −5.0%, d = −0.84), Pericyte cells (mean diff. = −0.8%, d = −0.76), and Adipocytes (mean diff. = −0.5%, d = −1.00)—exhibited trends toward greater abundance in HST cattle, as indicated by negative mean differences and medium-to-large effect sizes. The complete statistical results, including confidence intervals and effect sizes for all cell types, are provided in Table S4. CellChat analysis demonstrated differential intercellular communication patterns between breeds, with ANG cattle exhibiting enhanced FAP-1-FAP-1 interactions and HST cattle showing increased LEndoC-Adipocyte crosstalk (Figure 7F,G).

**Figure 8:**
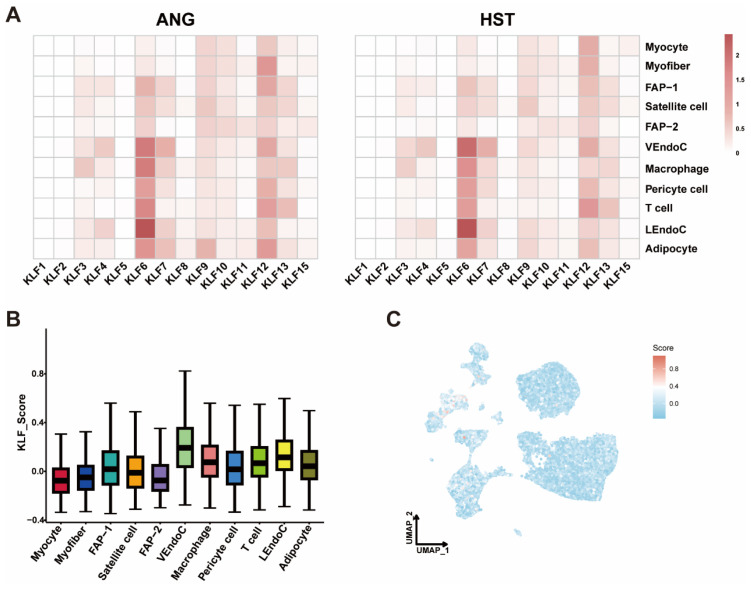
Expression of the *KLF* gene family in different cell types of LD. (**A**) The heatmap displays the expression of 14 *KLF* genes in 11 cell types of two cattle breeds. (**B**) The box plot displays the scores of 14 *KLF* genes merged into one gene set for each cell type. (**C**) The UMAP plot displays the results of the *KLF* gene set scoring mapping.

In order to analyze the cell-type-specific regulatory mechanism of the *KLF* transcription factor family in the bovine LD microenvironment and reveal the molecular regulatory differences between meat breed (ANG) and dairy breed (HST), we conducted an analysis of the expression of the *KLF* gene family in 11 cell types of ANG and HST cattle LD (Figure 8A). Overall, there was no heterogeneity in the expression of the *KLF* gene family in different cell types of breeds, but there were differences in expression patterns. We found that *KLF6* had the highest expression level in LEndoC of two breeds, with higher expression in VEndoC of HST cattle than in ANG cattle. *KLF6* was also expressed higher in FAP, Satellite cell, Macrophage, Pericyte cell, T cell, and Adipocyte of ANG cattle than in HST cattle. *KLF1* and *KLF2* are expressed at low levels in all cell types of both varieties. The *KLF12* gene is also expressed in all cell types, and its expression is higher in Myofiber, FAP-1, VEndoC, Macrophage, Pericyte cell, LEndoC, and Adipocyte of ANG cattle than in HST cattle, while the opposite is true in other cell types. Please refer to Table S5 for detailed expression levels. Using the AddModule Score algorithm, we calculated the *KLF* scores for each cell type and merged 14 *KLF* genes into one gene set. Surprisingly, VEndoC had the highest *KLF* score, followed by macrophages and LEndoC, followed by T cells, while other cell types had lower scores (Figure 8B,C, Table S6).

### 3.7. SHAP Explains the Contribution of the KLF Gene

**Figure 9:**
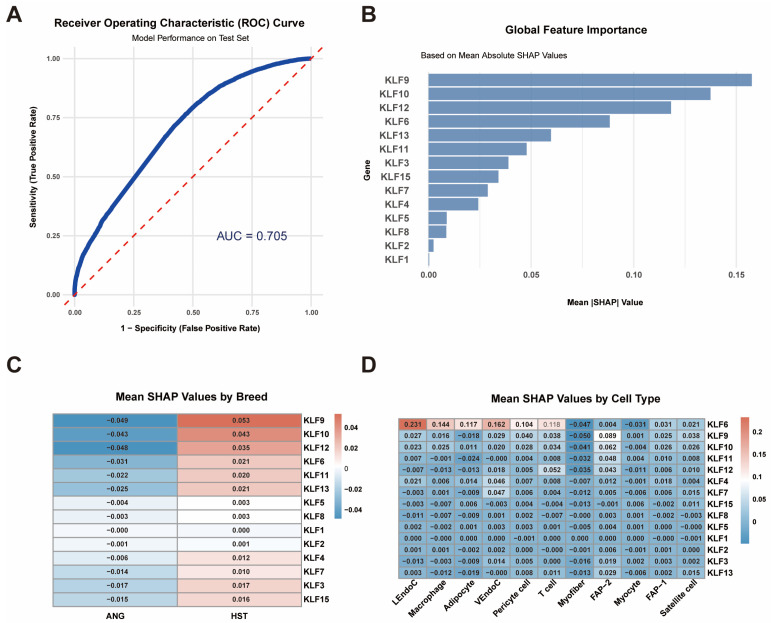
SHAP interpretation of the *KLF* gene. (**A**) The XGBoost classifier performance on test data is shown with a ROC curve (red line) and AUC value of 0.705. The dashed line represents the random guessing baseline (AUC = 0.5). (**B**) Bar plot showing mean absolute SHAP values for 14 *KLF* genes. (**C**) Heatmap comparing mean SHAP values between ANG and HST cattle. (**D**) Heatmap of mean SHAP values across 11 cell types.

To elucidate the regulatory roles of *KLF* genes in bovine muscle development, we performed SHAP analysis on snRNA-seq data from ANG and HST cattle (Figure 9). An XGBoost classifier achieved moderate predictive performance (AUC = 0.705, 95% CI: 0.68–0.73) for breed classification (Figure 9A). Global feature importance analysis revealed *KLF9*, *KLF10*, and *KLF12* as the top three contributors (mean|SHAP| = 0.16, 0.14, and 0.12, respectively), suggesting these genes drive transcriptional differences between breeds (Figure 9B, Table S7).

Heatmap visualization demonstrated breed-specific contribution patterns: *KLF* genes showed positive SHAP values (HST-enriched) versus negative values (ANG-enriched) across cell types (Figure 9C). Notably, *KLF6* exhibited the highest cell-type-specific contribution in LEndoC (mean SHAP = 0.231), exceeding other cell types by 2.3-fold (Figure 9D). Elevated *KLF6* contributions were also observed in macrophage (0.144), adipocyte (0.132), and VEndoC (0.118), correlating with its expression patterns identified in prior analyses.

## 4. Discussion

Over the past few decades, extensive research has been conducted on the biological functions of the *KLF* gene family in mammals, including roles in adipogenesis, myogenesis, tumorigenesis, and the regulation of cellular and tissue metabolism [31,32,33,34,35]. However, systematic research on the expression patterns and regulatory functions of the Bos taurus family in the complex cellular ecosystem of the muscle tissue microenvironment, an important agricultural species, is still relatively scarce. This study comprehensively identified the bovine *KLF* gene family for the first time by integrating bioinformatics, comparative genomics, and single-nuclei transcriptomics. The expression profiles of the *KLF* gene family in the longest dorsal muscle of ANG and HST cattle were plotted at single-nuclei resolution, revealing its expression heterogeneity, breed-specific regulatory patterns, and potential functions. This provides new insights into the role of the *KLF* family in cattle muscle development and meat quality trait formation.

The *KLF* family is a group of highly conserved zinc finger transcription factors that bind GC-rich DNA sequences and play pivotal roles in proliferation, differentiation, and metabolic regulation [36]. It is important to clarify that the conserved motifs identified de novo by MEME analysis in this study are distinct from the canonical C_2_H_2_ zinc finger DNA binding domain that defines the *KLF* family. The triple C_2_H_2_ domain is a well-characterized, conserved structural unit responsible for GC-rich DNA binding, and its presence was a prerequisite for classifying a protein as a *KLF* member (validated by our Pfam analysis). In contrast, the MEME-predicted motifs represent additional conserved sequence patterns outside the DNA-binding domain. These motifs may underlie novel functional divergences, such as differential protein–protein interaction or subcellular localization signals that contribute to the functional specialization of *KLF* members across evolutionary lineages and cell types. Our integrated single-nuclei transcriptomic and machine learning analysis revealed the cell-type-specific regulatory landscape of *KLF* genes in bovine muscle, with *KLF6* emerging as a key player in endothelial and adipocyte populations. The high abundance of *KLF6* in adipocytes, particularly in the meat breed ANG, aligns with its well-established role as a positive regulator of adipocyte differentiation [37,38]. This suggests that its elevated expression may contribute to the enhanced intramuscular fat deposition (marbling) characteristic of Angus cattle. Furthermore, its significant expression in VEndoC and LEndoC resonates with its documented function in vascular remodeling [38], implying a potential role in modulating blood vessel and lymphatic network formation within the muscle microenvironment. The uniquely high SHAP value of *KLF6* specifically in LEndoC cells, far exceeding that in other types, strongly suggests a previously underappreciated and critical function in bovine lymphatic biology, potentially mediating immune cell trafficking or metabolic waste clearance in a breed-specific manner. On the contrary, *KLF12* is upregulated in multiple cell types such as myofiber and FAP-1 in ANG cattle. This cell-type-specific expression pattern strongly suggests that *KLF* members synergistically regulate processes such as muscle tissue development, immune microenvironment, angiogenesis, and fat deposition by exercising their functions in different cell populations.

An important aspect of this study is the integration of differential expression analysis with machine learning (XGBoost) to quantify the relative contributions of *KLF* genes in driving transcriptomic differences between breeds. SHAP analysis provides objective and quantitative evidence for identifying key regulatory factors. The global feature importance analysis consistently identified *KLF9*, *KLF10*, and *KLF12* as the main contributors to distinguishing ANG and HST cattle varieties, indicating that they play a central role in shaping variety-specific transcription programs. More importantly, the decomposition of SHAP values at the cell type level reveals an unprecedented fine regulatory landscape: *KLF6* exhibits highly specific contributions in lymphatic endothelial cells, with SHAP values far exceeding those of other cell types. This finding is consistent with the high expression level of *KLF6* in this cell type, strongly suggesting that *KLF6* may have a unique and critical regulatory function in bovine lymphatic endothelial cells, and may be a key molecular switch mediating immune microenvironment or lymphatic vessel function differences between breeds. Meanwhile, the significant contribution of *KLF6* in macrophages and adipocytes suggests that it may jointly affect the final meat quality traits by regulating the interactions of multiple cell types.

One of the key findings of this study is the revelation of the variety specificity of *KLF* gene expression and regulation. Cell population composition analysis showed that the proportion of myofiber in the meat breed ANG was significantly higher than that in the dairy breed HST, while HST had a richer matrix–cell population (such as FAPs). This difference in cell composition is likely the cellular basis for the differences in meat production performance and meat quality between the two varieties. SHAP analysis further supports this conclusion from the perspective of functional contribution, revealing the differential driving effects of different *KLF* genes on variety characteristics under different cellular backgrounds. These results indicate that the *KLF* family is not equally involved in regulation but rather dominated by a few key members in specific cell types, leading to genetic differences between varieties. This provides a potential molecular mechanism for explaining the phenotypic differences between ANG and HST in muscle growth and fat deposition.

Based on protein interaction network prediction, bovine KLF protein interacts with key transcription factors known to regulate muscle and fat development, such as *CEBPB* and *ESRRB*. Recently, in-depth research on skeletal muscle RNA-seq data has shown that *CEBPB* can serve as a biomarker related to fat generation in muscle aging [39]. *ESRRB* is also known to be an important TF for myogenic development and stem cell function, and studies have shown that *ESRRB* promotes the transformation of fibroblasts into induced myogenic stem cells [40]. This suggests that bovine *KLF* may form complexes with such core regulatory factors and integrate into the complex transcriptional network that regulates muscle development and metabolism, thereby affecting the final economic traits.

The conclusion of this study is mainly based on bioinformatics prediction and correlation analysis, and its specific molecular mechanism still needs to be verified through experiments. For example, the potential functional changes caused by motif deletion in *KLF2/8*, as well as the key regulatory functions suggested by the high SHAP value of *KLF6* in lymphatic endothelial cells, need to be further explored through functional experiments such as ChIP seq and CRISPR-Cas9 gene editing in vitro and in vivo. Furthermore, we note that our computational analysis did not include dedicated doublet detection or ambient RNA mitigation. While standard QC metrics (e.g., UMI/gene counts, mitochondrial percentage) were applied, future studies employing specialized tools (e.g., DoubletFinder, Scrublet, or DecontX) would help further refine the dataset and confirm the robustness of the findings, particularly in rare cell populations. In addition, this study focuses on a specific time point in adult cattle. If future research can cover multiple key developmental stages, it will be able to more dynamically reveal the regulatory role of the *KLF* family in the entire process of muscle development.

## 5. Conclusions

In summary, this study presents the first systematic single-nuclei atlas of the bovine *KLF* gene family within the skeletal muscle microenvironment. Our multi-omics approach, integrating comparative genomics and snRNA-seq data from over 110,000 high-quality nuclei, revealed cell-type-specific expression patterns and significant differential expression of *KLF* genes between meat (ANG) and dairy (HST) cattle breeds. Through explainable machine learning (XGBoost, AUC = 0.705), we quantitatively identified *KLF6*, *KLF9*, *KLF10*, and *KLF12* as top contributors to transcriptional differences between breeds, with *KLF6* demonstrating particular cell-type-specific regulatory potential in lymphatic endothelial cells. These statistically robust findings provide unprecedented insights into *KLF* functional diversity in bovine muscle biology and deliver high-confidence candidate targets for precision breeding. While this study establishes strong transcriptional associations, future research employing functional validation via CRISPR-Cas9 screening in bovine cellular models will be essential to confirm causal relationships and advance toward practical breeding applications.

## Figures and Tables

**Table 1:** *KLF* gene family information in Bovine (*B. taurus*).

Gene Name	Gene ID	Protein ID	CDS ID	Chr	Genomic Location	Exon	AA	Mass (Da)
KLF1	ENSBTAG00000006083	ENSBTAP00000043340	ENSBTAT00000046003	7	12,671,273–12,675,059	3	372	39,444
KLF2	ENSBTAG00000052504	ENSBTAP00000074931	ENSBTAT00000108559	7	6,685,278–6,687,052	2	78	8035
KLF3	ENSBTAG00000017488	ENSBTAP00000057281	ENSBTAT00000085769	6	57,957,697–57,974,562	5	346	38,943
KLF4	ENSBTAG00000020355	ENSBTAP00000072107	ENSBTAT00000077353	8	97,175,407–97,178,550	3	145	16,518
KLF5	ENSBTAG00000002129	ENSBTAP00000002751	ENSBTAT00000002751	12	47,799,775–47,817,130	4	544	50,477
KLF6	ENSBTAG00000015188	ENSBTAP00000020207	ENSBTAT00000020207	13	44,597,409–44,601,156	2	309	33,456
KLF7	ENSBTAG00000044097	ENSBTAP00000066321	ENSBTAT00000078679	2	95,426,838–95,439,630	2	300	32,220
KLF8	ENSBTAG00000005852	ENSBTAP00000067755	ENSBTAT00000071858	X	93,269,040–93,444,986	5	248	26,365
KLF9	ENSBTAG00000016229	ENSBTAP00000021593	ENSBTAT00000021593	8	46,587,347–46,611,150	2	244	27,219
KLF10	ENSBTAG00000014396	ENSBTAP00000067318	ENSBTAT00000076027	14	61,770,518–61,775,159	4	483	53,028
KLF11	ENSBTAG00000046218	ENSBTAP00000063288	ENSBTAT00000080078	11	87,549,525–87,558,666	4	523	55,735
KLF12	ENSBTAG00000044007	ENSBTAP00000053615	ENSBTAT00000061307	12	48,526,715–48,823,511	7	408	44,613
KLF13	ENSBTAG00000051090	ENSBTAP00000057849	ENSBTAT00000079732	21	27,672,129–27,709,397	2	296	31,917
KLF15	ENSBTAG00000008313	ENSBTAP00000063598	ENSBTAT00000125115	22	60,616,031–60,648,782	2	394	41,542

## Data Availability

The datasets presented in this article are not readily available because the data are part of an ongoing study.

## Supplementary Materials

The following supporting information can be downloaded at: https://www.mdpi.com/article/10.3390/ani15192930/s1, Table S1: Summary of sequencing statistics and quality metrics per sample; Table S2: UniProt accession IDs of KLF genes from 4 species; Table S3: Protein sequences of 64 KLF genes from 4 species; Table S4: Statistical Comparison of Cell Type Proportion Differences Between ANG and HST Breeds; Table S5: The average expression levels of 14 KLF genes in 11 cell types of two cattle breeds; Table S6: Scoring of KLF gene set in 11 cell types; Table S7: The average SHAP value of 14 KLF genes.

## Abbreviations

ANG	Angus
HST	Holstein
LEndoC	Lymphatic endothelial cells
VEndoC	Vascular endothelial cell
FAP	Fibro/Adipogenic Progenitor
snRNA-seq	Single-nucleus RNA sequencing
LD	Longissimus dorsi
SHAP	Shapley Additive exPlanations
KLF	Krüppel-like factor
PPI	Protein–protein interaction
